# Individualistic Population Responses of Five Frog Species in Two Changing Tropical Environments over Time

**DOI:** 10.1371/journal.pone.0098351

**Published:** 2014-05-30

**Authors:** Mason J. Ryan, Michael M. Fuller, Norman J. Scott, Joseph A. Cook, Steven Poe, Beatriz Willink, Gerardo Chaves, Federico Bolaños

**Affiliations:** 1 Department of Biology, University of New Mexico, Albuquerque, New Mexico United States of America; 2 Museum of Southwestern Biology, University of New Mexico, Albuquerque, New Mexico United States of America; 3 Escuela de Biología, Universidad de Costa Rica, San Pedro, San José, Costa Rica; Clemson University, United States of America

## Abstract

Roughly 40% of amphibian species are in decline with habitat loss, disease, and climate change being the most cited threats. Heterogeneity of extrinsic (e.g. climate) and intrinsic (e.g. local adaptations) factors across a species’ range should influence population response to climate change and other threats. Here we examine relative detectability changes for five direct-developing leaf litter frogs between 42-year sampling periods at one Lowland Tropical Forest site (51 m.a.s.l.) and one Premontane Wet Forest site (1100 m.a.s.l.) in southwest Costa Rica. We identify individualistic changes in relative detectability among populations between sampling periods at different elevations. Both common and rare species showed site-specific declines, and no species exhibited significant declines at both sites. Detection changes are correlated with changes in temperature, dry season rainfall, and leaf litter depth since1969. Our study species share Least Concern conservation status, life history traits, and close phylogenetic relationship, yet their populations changed individualistically both within and among species. These results counter current views of the uniformity or predictability of amphibian decline response and suggest additional complexity for conservation decisions.

## Introduction

A primary focus of community ecology is to understand how species respond to environmental variation across space and time [1]. This focus has gained urgency as anthropogenic pressures alter species dynamics [2], pushing many species toward extinction [3]. Long-term studies of community ecology can link shifting population patterns to changes in climate and inform conservation efforts for imperiled species and communities [4], [5].

Tropical amphibians are at the forefront of the current extinction crisis [4], [6]. As a consequence of climate change, habitat loss, disease, and interactions among these factors, 40% of the 7,125 known amphibian species are at high risk of extinction in the near future [6], [7]. Disease-induced population crashes caused by the fungal pathogen *Batrachochytrium dendrobatidis* (*Bd*) have been documented in many amphibian communities [8], but little is known about long-term population trends and non-disease threats in most species [4], [7].

Identifying decadal-scale population trends for tropical amphibians has been difficult due to a dearth of historical baseline population data [9], [10]. In one case where such data were available, an entire Neotropical leaf litter amphibian fauna was found to have declined over a 35-year period in the Atlantic lowlands of Costa Rica, with declines linked to climate change [11]. Although long-term site-specific studies provide valuable insights into local population responses to environmental change, they cannot assess interpopulation differences in susceptibility across species’ ranges [12], [13].

Measuring population changes in multiple environments is particularly important because climate change is not occurring uniformly across the landscape [14]. Instead, changes are localized due to variable conditions (e.g., topography, prevailing winds) resulting in a mosaic of novel climatic conditions at small spatial scales [14]–[16]. Species with broad ranges may include locally adapted populations that exhibit different tolerances to changing environmental conditions and disease [17]–[19]. Long-term assessments that incorporate population trends across more than one environment are needed to understand range-wide responses of species to change.

Characterizing interpopulation variation in the environmental sensitivity of amphibian species is critical for developing research and conservation priorities in a rapidly changing world [3], [12]. Amphibians are especially sensitive to long-term global climate change because warming temperatures and altered hydrologic cycles are expected to increase thermal stress [20], affect disease susceptibility [21], desiccate breeding habitats [22], reduce availability of critical microhabitats [11], [23], and alter foraging behavior and efficiency [24], [25]. Yet, because climate change is occurring heterogeneously across the landscape [14], it is unclear if all populations of a species are at equal risk [13].

In the Neotropics, frogs of the clade Terrarana dominate leaf litter vertebrate community diversity and abundance [26]–[28]. Terrarana frogs reproduce by direct development, have no association with aquatic habitats, and depend on standing leaf litter for most aspects of their life, e.g., refugia, foraging, and egg laying sites [28], [29]. Because of their strong leaf litter association and independence from aquatic habitats, many Terrarana frog species are expected to be directly or indirectly more vulnerable to climate change than disease [9], [11], [30]. There is a wide-range of *Bd*-susceptibility within Terrarana with riparian species being more susceptible to *Bd* compared to the terrestrial species studied here [30]–[32]. Following *Bd*-declines some strictly terrestrial Terrarana species increase in abundance and become dominant components of post-*Bd* assemblages [30]. By studying non-*Bd* vulnerable frog species it is possible to evaluate the effects of environmental change on the remaining species in a post-*Bd* world.

Here we expand the growing field of longitudinal population comparisons (e.g., [11], [33], [34]) by studying five wide–ranging Terrarana leaf litter frog species in two distinct tropical environments. The broad geographic distributions, syntopy, close phylogenetic relationship, and ecological similarities of these species make them ideal for comparative exploration of long–term population changes. We address relative detection changes within and among species, both within and between environments and over time. We used plot presence/absence data as an assay of relative detection probability from 1969 and 2009–2012 from one Lowland Tropical Forest (51 m.a.s.l.) and one Premontane Wet Forest (1100 m.a.s.l.) environment in southwestern Costa Rica.

## Methods

### Ethics Statement

The study was approved and conducted under animal care protocol 08UNM041 by the Institutional Animal Care and Use Committee at the University of New Mexico. Costa Rica Research Permits were granted through Javier Guevara at Ministerio de Ambiente y Energia (MINAE) to MJR. This study did not involve any endangered species.

### Study Sites and Field Methods

Las Cruces Biological Station (LCBS) protects approximately 227 hectares of Premontane Wet Forest in the Coto Brus Valley (8.785778 N; 82.958889 W Decimal Degrees; 1100 m elevation) on the Pacific versant of the southern Talamanca Mountains, Puntarenas Province, Costa Rica. The 39–year mean annual rainfall is 3442 mm, with a distinct dry season from January–March, and a mean annual temperature of 20.7°C (Table S1; [35]). LCBS has been protected since 1962 and is surrounded by a matrix of smaller fragments and agricultural land [36].

Fundación Neotropica Station is located ∼2 km southwest of Rincón de Osa (Rincón; 8.69602 N, – 83.50139 W, 51 m) on the Osa Peninsula in the southwest Pacific lowlands, Puntarenas Province, Costa Rica within the lowland Tropical Forest Zone. The region was forested until the late 1960s when a logging camp was established, and by the 1980s deforestation was nearly complete [37]. By 1996 the flatlands had been converted to pastureland, but the adjacent foothills and steep slopes remain largely forested, including approximately 300 hectares of primary and older secondary forest [37]. Because our surveys require relatively flat forest (see below), all studied plots were in the foothills of the forested mountain, approximately 200 m from cattle pastures. Plots were on the Fundación Neotropica Station or within 1 km of the station on adjacent private property. The 52–year mean annual rainfall for Rincón region is 4730 mm with a distinct dry season from January–March, and a mean annual temperature of 27.5°C (Table S1; [38]).

Participation of the original researcher, Norman J Scott, allowed us to replicate the field data collection and plot set-up techniques used for the 1969 baselines. Each plot was 25-foot square. The original plots were not resampled and each year new plots were sampled to minimize possible impacts from litter removal disturbances during sampling. Plots were placed haphazardly within the forest in flat areas away from trails, tree fall gaps and slopes; we could not completely randomize plot placement within the forest patches. We used Scott’s [26] clearing techniques that require removal of all leaf litter to maximize frog observations per plot. After plots were sampled the leaf litter and debris were added back to the plots. During the dry season of March 1969, Scott [26] sampled 10 plots: five at LCBS and five at Rincón; from 2009–2012 we sampled 78 plots, 38 at LCBS and 40 at Rincón in March. We measured leaf litter depth using a ruler at the corners and center of each plot and averaged these measurements for an estimate of leaf litter depth/plot.

### Statistical Methods for Relative Detection Probability

We measured changes in detection probability by scoring the proportion of plots occupied, e.g. presence/absence, for each species during a given sampling period (Summarized in Table S1). This is a statistically simplified approach to estimating detection probability, and we refer to this as relative detection probability. We used this approach because of limitations imposed by the original study design that precluded the use of robust algorithm based detection probability approaches such as Program PRESENCE [38]. Algorithm based methodologies have specific assumptions in model building that include primary and secondary sampling periods per field season [39]. Our study does not meet these assumptions because we lack a secondary sampling period. Instead, we attempted to exactly replicate the original study, which did not have a secondary sampling period [26].

Although we also collected abundance data, we analyzed relative detection probability rather than abundance because four of our five focal species were present in low numbers (see below) and such over-dispersion may cause problems for non-logistic approaches [40]. To assess changes in species presence/absence over time, we regarded 1969 as the initial sample period, and all samples recorded between 2009 and 2012 as secondary measurements of a repeated measures experiment. We compared the 1969 samples to the later period formed by pooling the 2009–2012 samples. We investigated temporal changes in presence/absence at each site separately and jointly (e.g., by including a time x site interaction term).

We converted the raw species counts recorded from each plot to presence/absence data, and analyzed the resulting occupancies by logistic regression. Statistical modeling was hindered by the condition of the data, which was characterized by low initial samples size and sparse occupancy (i.e., many empty samples). In addition, the repeated measures design, and probable spatial autocorrelation of samples collected from the same site [41], raised concerns that residual errors may be spatially and temporally correlated. The aforementioned data issues are problematic for standard logistic methods, which rely on maximum likelihood calculations to estimate model parameters. The Firth logistic method, which uses a penalized likelihood method, was developed to overcome computational challenges presented by small sample sizes, data sparsity, and non-independence [42]. Therefore, all of our logistic modeling was performed using the Firth method.

In evaluating the contribution of Site and Period to presence/absence, we constructed a separate model for each species. We considered an effect to be statistically significant if the probability of a non-zero coefficient (i.e., alpha) was less than 0.05. All statistical modeling was performed in R [43].

The year 2011 was a strong La Niña year, which resulted in significantly higher than normal rainfall in lower Central America [44] and at our study sites (unpublished data). The intent of our study was to focus on average trends and not exceptional events such as the strong La Niña. We included 2011 in preliminary analyses (which bolstered our current conclusions of population decline; see below), but because it differed from the other recent sampling years we excluded it from analyses presented here due to concerns of conflating long-term trends with changes due to anomalous climatic effects [44].

### Climate Trends

We used meteorological data from the Loma Linda and LCBS meteorological stations to explore long-term climate patterns at LCBS. The Loma Linda (8.7385 N; – 82.922717; 1100 m) station is located 14 km south of LCBS and includes rainfall and temperature data from 1973–2007. The LCBS station has rainfall and temperature data from 2005–2012. Loma Linda and LCBS had comparable weather for the three years of data shared by these stations (2005–2007; Fig. S1 and S2). Combining data for these stations provided a continuous record from 1973–2012.

There are no complete and reliable meteorological records from any weather station in the vicinity of Rincón. To reconstruct the recent historical temperature and rainfall profile we relied on meteorological data from three regional lowland weather stations. Hacienda Barú National Wildlife Refuge (N 9.27152; – 83.88162 W; 24 m) is located 45 km north of Rincón and has documented monthly rainfall from 1981–2011 and temperature from 2001–2011. The Golfito weather station (8.39 N; – 83.11 W; 15 m) of the Instituto Meteorológico Nacional is located approximately 25 km south of Rincón and has kept monthly rainfall records from 1960–1983. We determined that Hacienda Barú and Golfito receive similar rainfall using the same methods as above for the four years of overlap, 1980–1983 (Fig. S3). The third station is in David, Panama (8.4 N, – 82.424167 W; 27 m) 117 km east of Rincón and the closest Pacific lowland station with long-term monthly temperature data from 1973–2000 [16]. We combined the weather data from these three stations to reconstruct general decadal climate change for the Pacific lowlands near Rincón [16].

We used linear regression to explore long-term changes in rainfall and temperature at each site. The binned wet and dry season rainfall and temperature data allowed us to test long-term, seasonal rainfall and temperature trends. We follow McDiarmid and Savage [38] in classifying months with less than 200 mm of precipitation as dry season (i.e., January–March).

## Results

### Changes in Relative Detection Probability

We were able to calculate relative detection probability changes for all five species from the plot presence/absence proportions for 1969 and 2009–2012 (excluding 2011) (Table 1). The general Firth logistic regression model results show that relative detection probability significantly changed for three species between sampling periods and one species showed a significant time Period X Site interaction (Table 2).

**Table 1:** Plot presence/absence for 1969 and 2000s sampling periods.

Species	LCBS 1969	LCBS 2000s	Rincón 1969	Rincón 2000s
*Craugastor crassidigitus*	2/5 [20%]	9/28 [32%]	4/5 [80%]	1/30 [3%]
*Craugastor rugosus*	2/5 [20%]	0/28 [0%]	1/5 [20%]	0/30 [0%]
*Craugastor stejnegerianus*	5/5 [100%]	17/28 [60%]	5/5 [100%]	27/30 [90%]
*Diasporus vocator*	3/5 [60%]	0/28 [0%]	2/5 [40%]	2/30 [6%]
*Pristimantis ridens*	4/5 [80%]	4/28 [14%]	1/5 [20%]	5/30 [16%]

Proportions of plots occupied by each species at LCBS and Rincón between sampling periods. Values in brackets are proportion of plots occupied during that sampling period. These data were used to calculate relative detection probabilities used in the logistic regression analysis. See Table S1 for presence/absence for each individual year.

**Table 2:** General logistic regression results.

Species	TimePeriod*P*-value	Coefficient ±STD Error	Site*P*-value	Coefficient± STD Error	Time PeriodX Site*P*-value	Coefficient± STD Error
*Craugastor crassidigitus*	0.082	−0.23±0.91	0.236	1.44±1.37	0.032*	−2.93±1.54
*Craugastor rugosus*	0.007**	−3.71±1.71	0.548	−0.76±1.37	0.770	0.69±2.47
*Craugastor stejnegerianus*	0.106	−1.98±1.66	1.00	0.00±2.29	0.468	1.64±2.39
*Diasporus vocator*	0.007**	−3.71±1.71	1.00	0.00±1.28	0.385	1.60±2.05
*Pristimantis ridens*	0.004**	−2.79±1.16	0.076	−2.20±1.46	0.099	2.36±1.62

Logistic regression results relative detection probability calculated from the plot occupancy.

See text for details on analyses. Significance levels: * = 0.05; ** = 0.01.

**Figure 1:**
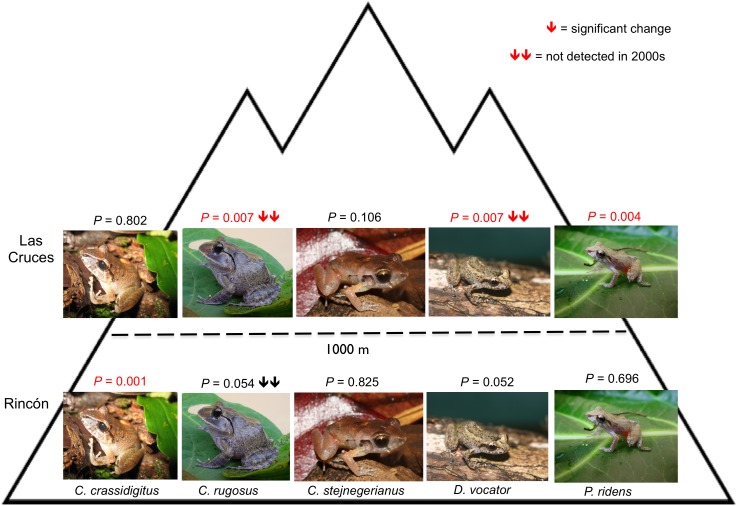
Changes in detection of the five species between the two sites. The site-specific Firth logistic regression *P*–values and direction of relative detection changes between sampling periods and elevation for each species. Upper row is LCBS and lower row is Rincón. See text for details of analysis and Table 1 for raw data.

The site-specific Firth logistic regression results show individualistic changes in relative detection among species and sites. One species, *Craugastor stejnegerianus,* showed no change in relative detection at both sites over time, and the other species exhibited unique changes between sites over time (Table 3, Fig. 1). Three of five species showed a negative change in relative detection at the mid-elevation site, LCBS; two of these species, *C. rugosus* and *Diasporus vocator*, can be considered rare in our samples and were not detected in the later sampling period. At Rincón, two species showed a negative change, *C. crassidigitus* and *C. rugosus; C. rugosus* was rare in our samples and was not detected during the later sampling period (Fig. 1).

**Table 3:** Firth logistic regression results.

Species	Rincón *P*-value	Coefficient ± STD Error	LCBS *P*-value	Coefficient ± STD Error
*Craugastor crassidigitus*	0.001**	−3.16±1.18	0.802	−0.23±0.99
*Craugastor rugosus*	0.054	−3.01±1.78	0.007**	−3.71±1.71
*Craugastor stejnegerianus*	0.825	−0.34±1.72	0.106	−1.98±166
*Diasporus vocator*	0.052	−2.10±1.13	0.007**	−3.71±1.71
*Pristimantis ridens*	0.696	−0.44±1.14	0.004**	−2.79±1.16

Site-specific results of Firth logistic regression between sampling periods.

**denotes 0.01 significance level.

### Climate Variables

**Figure 2:**
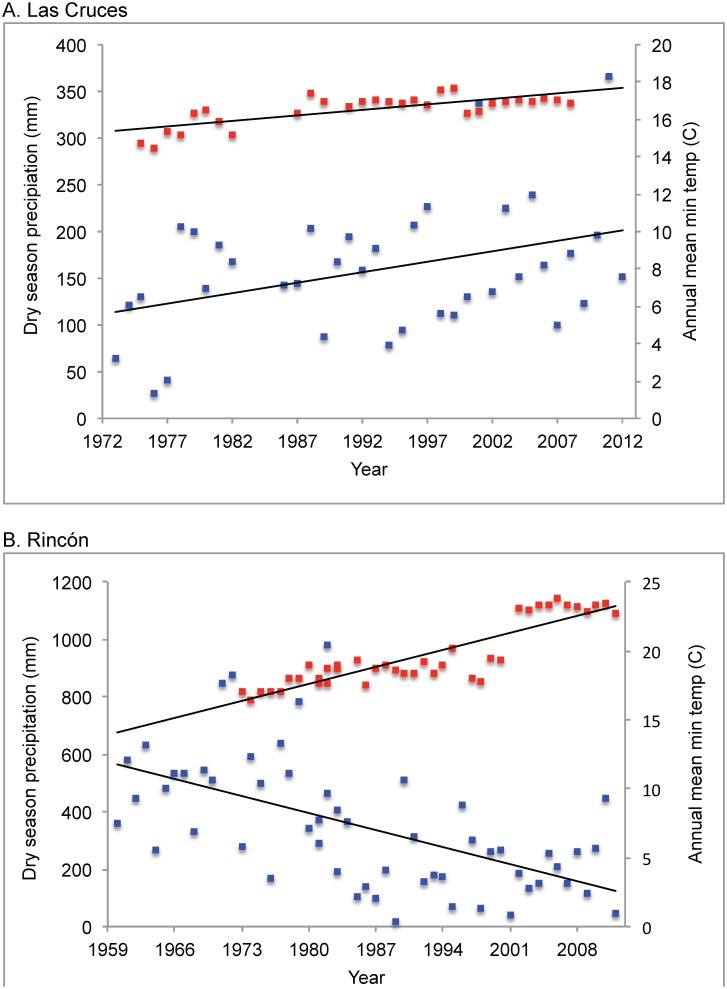
Climatic changes for LCBS and Rincón. Long-term trends in dry season precipitation and minimum annual temperature for A) LCBS and B) Rincón. Red squares represent temperature; blue squares represent dry season rainfall.

We observed a significant increase in annual mean minimum temperature at Rincón of 0.059°C/year (R^2^ = 0.558; *P* = 0.0001) and at LCBS of 0.064°C/year (R^2^ = 0.420; *P* = 0.0001) since 1973 (Fig. 2). We found dry season precipitation changed divergently for each elevation. At Rincón, dry season precipitation significantly decreased by 8.47 mm/year on average since 1960 (R^2^ = 0.324; DF = 55; *P* = 0.0001). There was no significant change in wet season (R^2^ = 0.044; DF = 55; *P* = 0.129) or annual precipitation (R^2^ = 0.006; DF = 55; *P* = 0.554). Conversely, at LCBS dry season precipitation significantly increased by 2.24 mm/year on average since 1973 (R^2^ = 0.148; DF = 34; *P* = 0.018) with no significant change in wet season (R^2^ = 0.028; DF = 34; *P* = 0.339) or annual precipitation (R^2^ = 0.042; DF = 34; *P* = 0.242).

### Litter Depth

At LCBS mean leaf litter depth decreased significantly from 7.02±2.11 cm (Std Dev) in 1969 to 4.88±1.69 cm in the 2000s (Kruskal-Wallis χ^2^ = 4.425; DF = 1; *P* = 0.037). At Rincón mean leaf litter depth exhibited no significant change between March 1969 and the 2000s (Kruskal-Wallis χ^2^ = 1.553; DF = 1; *P* = 0.219).

## Discussion

Relative detection probability changes in the five frog species indicate two broad patterns in time and space that are associated with substantial changes in temperature, dry season precipitation and leaf litter depth. First, the site-specific results show relative detectability changes varied among species between sites, with neither site exhibiting uniform declines across all species between 42–year sampling periods (Fig. 1). Second, we observed substantial intraspecific variation in different environments between 42-year sampling periods. Our results suggest that these widely distributed leaf litter frogs show individualistic responses to environmental change, a pattern that fits the individualistic or Gleasonian ecological view [12], [45].

The scope, design and results of our study differ from previous tropical amphibian decline studies in three main ways. First, previous amphibian population change studies documented community–wide declines within a single locality and environment (i.e. lowland rainforest or montane cloud forest) [11], [46], [47]. Our study measured simultaneous changes in the same five species across two distinct environments over time and found no uniform, community-wide decline. Our approach assessed inter- and intraspecific responses at broader geographic scales than previous work. Second, previous mid-elevation amphibian decline studies have focused on highly *Bd*-vulnerable riparian species that quickly declined (i.e., [30], [47], [48]) rather than terrestrial leaf litter species that are likely to be more vulnerable to climate change than *Bd* [9], [11], [30]. Our focus on non-*Bd*-vulnerable leaf litter frogs, 20-years after *Bd* arrived in the region (e.g. early 1990’s) (32), allowed us to investigate responses associated with climate change rather than the confounding or direct effects of disease. Third, we rely on changes in relative detection, instead of the more commonly used count data [11], [48], to measure if species have become more rare over time. This approach is more conservative than using count data to assess population change because it is less vulnerable to overestimating the magnitude of change for species with low sample sizes [49].

Amphibian populations are susceptible to stochastic variation and distinguishing natural amphibian population fluctuations from directional short- or long-term declines has been problematic [50], [51]. Extreme population fluctuations tend to be driven by unpredictable changes to aquatic breeding habitats, droughts, or deluges, impacting aquatic breeding species more than terrestrial breeding species [51], [52]. With no ties to running or standing water for reproduction, Terrarana frogs are not expected to exhibit short-term population fluctuations characteristic of aquatic species [11], [52]. Because the sampling years in our analysis represent periods of relatively constant weather (Fig. 2) it is unlikely our patterns reflect short-term changes. During the 2011 sampling year that we removed from analysis, both sites received approximately 50% more rainfall than the long-term means. We excluded this year in order to maintain comparability with the early (1969) sampling period, which received an unexceptional amount of rain.

### Site-specific Patterns

Because our study species are phylogenetically closely related and ecologically uniform, similar population responses to environmental change might be expected [30], [53]. Yet we observed individualistic responses among species, with no general community wide trends at either site despite significant changes in temperature and dry season rainfall (Fig. 2). This result is consistent with patterns observed in North American small mammals and birds. Moritz et al [33] found elevational range shifts to be variable among small mammal congeners over a 100-year period [33]. Taper et al. [12] found differing patterns of decline among species of insectivorous songbirds. These examples of species-specific responses highlight the complexity of predicting individual species and community responses to climate change.

Why did we observe species-specific responses among closely related, ecologically similar frog species? We can only speculate on mechanisms. Body size is an important predictor of species extinction threat from disease or climate change with large species at greater risk than small species [32], [54], but there is no relationship of body size and population trends among our species (Fig. 2, Table 1). We hypothesize that undetected micro–ecological differences (breeding phenology, diet, etc.) will emerge with future study of these species. For example, *Diasporus vocator* may have declined at LCBS because increased dry season rainfall saturated the soil leading to high rates of egg mortality [55]. Increased dry season rainfall was not evident at Rincón, and perhaps the eggs of *D. vocator* are especially sensitive to changes in rainfall patterns. This scenario is speculative, as we do not understand the ecology of *D. vocator* at this fine level. Autecological studies of our five frog species are needed to shed light on their varying responses.

### Intraspecific Patterns

Observed differences in relative detection changes within species show that these leaf litter frogs respond to long-term environmental change individualistically. The majority of our species showed a decrease in detection at one site but not the other site between the 42-year sampling periods (Fig. 2). A similar result has been found in birds, where 77% of 47 species that occurred in more than one environment varied in degree of population change between environments [12].

These individualistic responses could be attributed to varying environmental stressors at each site differentially affecting traits that are constant across species (see previous section). Alternatively, local variation in decline susceptibility within species may produce our observed patterns. For example, *Craugastor crassidigitus* declined at Rincón but not LCBS. Temperature increased significantly at both sites, so perhaps the population of *C. crassidigitus* at Rincón is sensitive to warmer temperatures but the population of *C. crassidigitus* at LCBS is tolerant to increased temperatures. Such a scenario likely oversimplifies the complexities of ecological interactions that may be operating. Additional factors such as invertebrate predation [56] and physiological stresses associated with increased temperatures and altered rainfall patterns [20], [57], or other dynamic interactions, may drive local changes. Regardless of the mechanisms, intraspecific variation in response to local environmental change is evident among both common and rare species.

One species, *Craugastor rugosus,* was rare at both sites in 1969 and not detected during the later sampling period (Table 1) and warrants special consideration. Because we did not detect *C. rugosus* during the later sampling period, we would infer that this species was locally extirpated. However, we conducted transect surveys to supplement the plot method and detected *C. rugosus.* Detection of this species using a secondary method contradicts the inference from our plot data. We suggest that when replicating historical population comparisons to assess declines, alternative survey methods should be employed to detect rare species. Such multifaceted approaches are especially needed when assessing population extirpations. Reliance on a single field survey method may overestimate a species’ threatened status.

Finally, due to the ubiquity of *Bd* in Costa Rica and it’s role in amphibian declines we cannot rule out the possibility that at least some of our observed changes are related to disease. This is more of a concern at LCBS than Rincón because no severe *Bd*-declines and die-offs have been reported at tropical lowland sites, despite *Bd* being detected at low elevations [58], [59]. *Bd* arrived in the LCBS region in 1993, almost 20-years before our later sampling period [32]. There is no rigorous documentation of the *Bd* die-off at LCBS, but many Bd-susceptible species such as *Atelopus varius*, *Craugastor ranoides*, and others are now absent from the frog fauna [60]. It is not possible for us to determine whether *Bd* played a role in the decline of our study species at LCBS. However, we note that Picco and Collins [61] detected *Bd* at LCBS but did not detect *Bd* on any strictly terrestrial, direct-developing frog species there, including two of our study species. Furthermore, our study species and their relatives are known to increase in community dominance [30] and abundance [62] within four years of *Bd*-related faunal collapse. We suspect that *Bd* has had an impact on many amphibian species at LCBS. However there is no evidence that any of our study species has been affected.

## Conclusions

We documented individualistic changes of frog populations between a 42–year period at two distinct sites. Observed changes are associated with increased temperatures, altered dry season rainfall, and changed leaf litter depth, all of which influence leaf litter amphibian populations [7], [11].

Increased rarity in some populations and not others is both troubling and optimistic in terms of long-term persistence of these leaf litter frogs. Our results are optimistic because we did not observe local extirpations of rare or common species despite decades of environmental change and disease emergence. Instead, common species have remained relatively common and rare species have remained rare, albeit at much lower detectability than in the past. This result is not consistent with other Neotropical studies that found declines and extirpations in both common and rare species [11], [46], [48]. On the other hand, these results are troubling because we detected declines in species of Least Concern not previously reported to have declined [6]. All of our study species have been categorized as Least Concern by the International Union for Conservation of Nature [6]. Population declines in species of Least Concern may be subtler than those of endangered species [63]. The apparently slow population attrition we detected contrasts with the rapid population crashes characteristic of many endangered forms [48], [64].

The above concerns clearly are pertinent to conservation decisions, but policy implications are not straightforward. Perhaps instead of focusing on a species as a whole, conservation actions should address local, geographically threatened or declining populations. The complicating factors we have identified, including decline of some populations of Least Concern species, interspecific variation among ostensibly ecologically uniform species, and differing intraspecific responses across space and time, should provide ample material for future discussions of conservation.

## Supporting Information

Figure S1 **Cumulative monthly rainfall for Las Cruces and Loma Linda weather stations between January 2005–December 2007, the only overlapping years of data collection.**
(TIFF)

Figure S2 **Mean minimum and maximum temperatures for Las Cruces and Loma Linda weather stations from January 2005–December 2007.**
(TIFF)

Figure S3 **Cumulative monthly rainfall for Golfito and Hacienda Baru weather stations between January 1981–December 1983, the only overlapping years of data collection.** The break in the Golfito line occurs because there was no rainfall value for October 1982.
(TIFF)

Table S1 **Proportion of plots occupied for each species by year and site.**
(DOCX)

Table S2 **Mean rainfall±standard deviation and duration of records for weather stations used in rainfall analysis.**
(DOCX)
