# Supplementary figures and images for: Individualistic Population Responses of Five Frog Species in Two Changing Tropical Environments over Time
Source: PLoS One. 2014 May 30;9(5):e98351. doi: 10.1371/journal.pone.0098351 (PMC4039490; doi:10.1371/journal.pone.0098351)

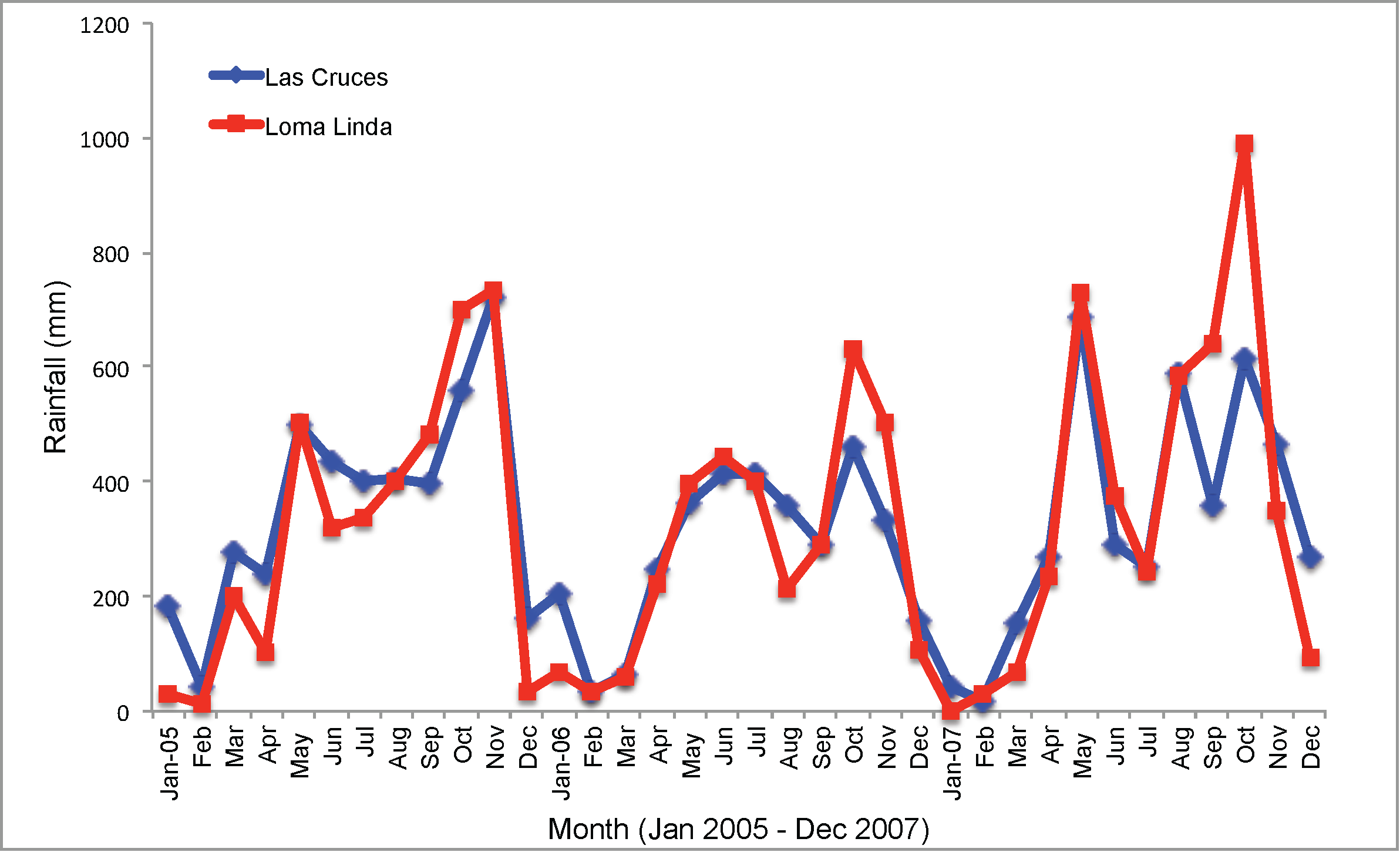

Supplement: Figure S1 — Cumulative monthly rainfall for Las Cruces and Loma Linda weather stations between January 2005–December 2007, the only overlapping years of data collection. (TIFF) [file pone.0098351.s001.tiff]

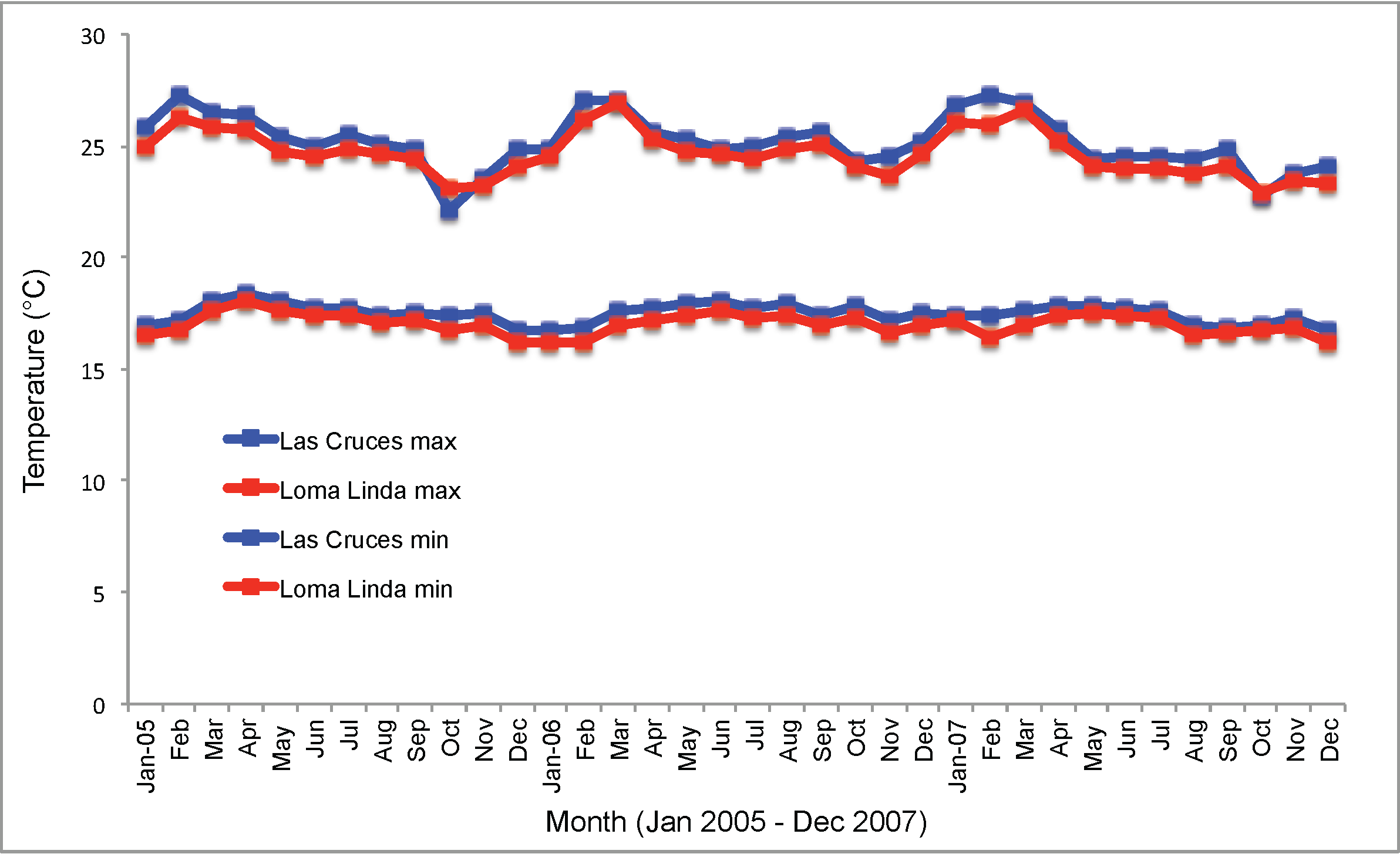

Supplement: Figure S2 — Mean minimum and maximum temperatures for Las Cruces and Loma Linda weather stations from January 2005–December 2007. (TIFF) [file pone.0098351.s002.tiff]

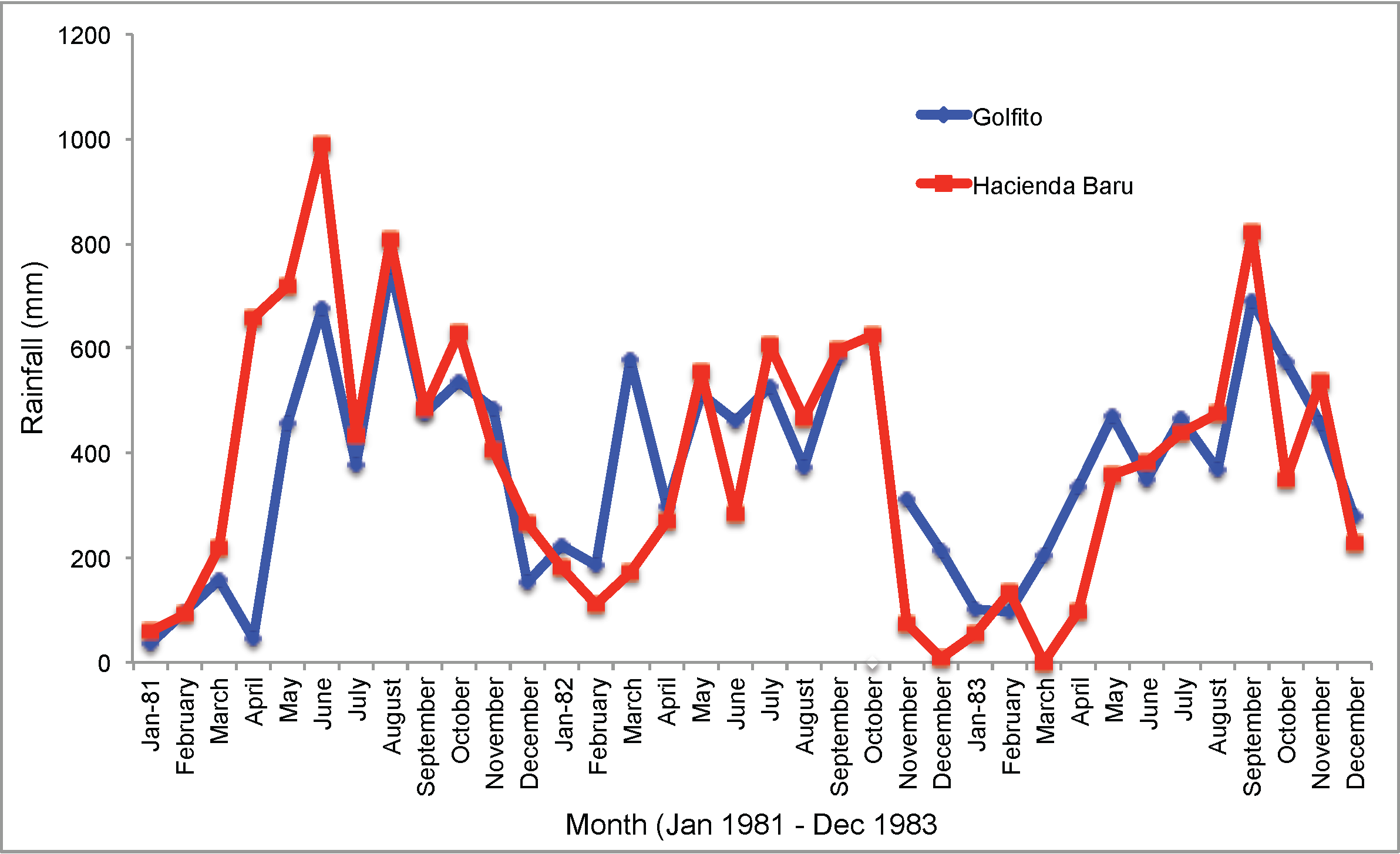

Supplement: Figure S3 — Cumulative monthly rainfall for Golfito and Hacienda Baru weather stations between January 1981–December 1983, the only overlapping years of data collection. The break in the Golfito line occurs because there was no rainfall value for October 1982. (TIFF) [file pone.0098351.s003.tiff]
