# Supplementary material for: Individualistic Population Responses of Five Frog Species in Two Changing Tropical Environments over Time
Source: PLoS One. 2014 May 30;9(5):e98351. doi: 10.1371/journal.pone.0098351 (PMC4039490; doi:10.1371/journal.pone.0098351)
Supplement: Table S1 — Proportion of plots occupied for each species by year and site. (DOCX) [file pone.0098351.s004.docx]

Table S1. Proportion of plots occupied for each species by year and site.

| Species | Site | 1969 | 2009 | 2010 | 2011 | 2012 |
| --- | --- | --- | --- | --- | --- | --- |
| *Craugastor*  *stejnegerianus* | LCBS | 5/5 (100%) | 7/10 (70%) | 6/10 (60%) | 3/10 (30%) | 4/8 (50%) |
|  | Rincón | 5/5 (100%) | 10/10 (100%) | 8/10 (80%) | 5/10 (50%) | 8/10 (80%) |
| *C. crassidigitus* | LCBS | 2/5 (40%) | 6/10 (60%) | 4/10 (40%) | 1/10 (0%) | 0/8 (0%) |
|  | Rincón | 3/5 (60%) | 1/10 (10%) | 0/10 (0%) | 0/10 (0%) | 1/10 (10%) |
| *C. rugosus* | LCBS | 2/5 (40%) | 0/10 (0%) | 0/10 (0%) | 0/10 (0%) | 0/8 (0%) |
|  | Rincón | 1/5 (20%) | 0/10 (0%) | 0/10 (0%) | 0/10 (0%) | 0/8 (0%) |
| *Diasporus vocator* | LCBS | 2/5 (40%) | 0/10 (0%) | 0/10 (0%) | 0/10 (0%) | 0/8 (0%) |
|  | Rincón | 2/5 (40%) | 1/10 (10%) | 1/10 (10%) | 2/10 (20%) | 0/8 (0%) |
| *Pristimantis ridens* | LCBS | 4/5 (80%) | 2/10 (20%) | 2/10 (20%) | 1/10 (10%) | 0/8 (0%) |
|  | Rincón | 1/5 (20%) | 2/10 (20%) | 0/10 (0%) | 1/10 (10%) | 3/10 (30% |
