# Supplementary material for: Individualistic Population Responses of Five Frog Species in Two Changing Tropical Environments over Time
Source: PLoS One. 2014 May 30;9(5):e98351. doi: 10.1371/journal.pone.0098351 (PMC4039490; doi:10.1371/journal.pone.0098351)
Supplement: Table S2 — Mean rainfall±standard deviation and duration of records for weather stations used in rainfall analysis. (DOCX) [file pone.0098351.s005.docx]

Table S2. Mean rainfall ± standard deviation local weather stations near the two study sites.

| Site | Mean Annual Rainfall | Mean Dry Season Rainfall | Mean Wet Season Rainfall | Duration |
| --- | --- | --- | --- | --- |
| **Las Cruces** |  |  |  |  |
| Loma Linda | 3468.5 ± 736.3 | 152.3 ± 66.0 | 3317.2 ± 734.4 | 1973—2007 |
| LCBS | 3959.5 ± 712.1 | 208.2 ± 77.0 | 3751.2 ± 687.8 | 2005—2012 |
| Combined | 3442.0 ± 749.2 | 163.6 ± 69.2 | 3282.4 ± 728.1 | 1973—2012 |
| **Rincón de Osa** |  |  |  |  |
| Golfito Station | 5006.1 ± 632.3 | 519.2 ± 205.0 | 4507.0 ± 570.8 | 1960—1983 |
| Hacienda Baru | 4518.5 ± 996.8 | 216.6 ± 131.0 | 4282.8 ± 978.7 | 1981—2012 |
| Combined | 4730.1 ± 884.8 | 346.3 ± 223.8 | 4385.8 ± 826.7 | 1960—2012 |
